# Analysis of surface temperature variation of lakes in China using MODIS land surface temperature data

**DOI:** 10.1038/s41598-022-06363-9

**Published:** 2022-02-14

**Authors:** Cong Xie, Xin Zhang, Long Zhuang, Ruixi Zhu, Jie Guo

**Affiliations:** 1 Nanjing Research Institute of Electronics Technology, Nanjing, 210000 China; 2 Information Center of Jiangsu Academy of Agricultural Sciences, Nanjing, 210000 China

**Keywords:** Environmental sciences, Hydrology

## Abstract

China has a great wealth of lake resources over a great spatial extent and these lakes are highly sensitive to climate changes through their heat and water budgets. However, little is known about the changes in lake surface water temperature (LSWT) across China under the climate warming conditions over the past few decades. In this study, MODIS land surface temperature (LST) data were used to examine the spatial and temporal (diurnal, intra-annual, and inter-annual) variations in LSWT of China’s lakes during 2001**–**2016. Our results indicated that 169 large lakes included in the study exhibited an overall increasing trend in LSWT, with an average rate of 0.26 °C/decade. The increasing rate of nighttime LSWT is 0.31 °C/decade, faster than that of daytime temperature (0.21 °C/decade). Overall, 121 (71.6%) lakes showed an increase in daytime temperature with a mean rate of 0.38 °C/decade, while the rest 48 (28.4%) lakes decreased in temperature with a mean rate of − 0.21 °C/decade. We also quantitatively analyzed the relationship of the lake surface temperature and diurnal temperature differences (DTDs) with geographical location, topography, and lake morphometry by utilizing multivariate regression analysis. Our analysis suggested that the geographical location (latitude and longitude) and topography (altitude) were primary driving factors in explaining the national lake water temperature variation (*P* < 0.001), which were also mediated by morphometric factors such as lake surface area and volume. Moreover, the diurnal lake temperature variations were significantly correlated with altitude, latitude, and lake surface area (R^2^ = 0.426, *P* < 0.001). Correlation analyses of LSWT trend and air temperature trend for each lake indicated that LSWT was positively correlated with air temperature in both daytime and nighttime for most lakes.

## Introduction

Lakes play an important role in ecological and environmental issues, and act as sentinels and integrators for the impacts of climate change on aquatic ecosystems^1^. In the context of global warming, lakes are undergoing rapid changes in physical, chemical and biological processes, which exert a considerable influence on the regional environment through the heat and water budgets. Lake surface water temperature (LSWT) is a critical physical parameter in the process of heat balance and moisture exchange between lake surfaces and the surrounding environment^2^, and influences both the limnological processes inside a lake and regional climate^3^. Investigations of the long-term changes in thermal structure of lakes are essential for providing fundamental knowledge for understanding lake ecosystem response to climate change^4^. Under current climate warming scenario, recent studies have reported that large lakes around the globe have showed an overall rapid warming trend, while the lake surface temperatures at regional scale have showed various degrees of variation^5,6^. The observed rapid and highly variable warming of lake surface water globally signals an urgent need to investigate the detailed variations of surface water temperature of lakes at a regional scale.

Over the past several decades, annual mean surface air temperature over China has experienced a rapidly warming trend based on measurements from meteorological stations^7,8^, which imposed significant impacts on inland water resources in China. According to the water resource investigations conducted since the 1950s, China has 2928 lakes with surface area of greater than 1 km^2^^9,10^. Lakes are dynamic and complex aquatic ecosystems, holding indispensable ecological values and providing aquatic habitats for plants, fish, waterfowl, and other animals. Under the influence of various climatic and socioeconomic conditions in China, these lakes have experienced complex physical and ecological changes due to climate variation and anthropogenic activity^11^. As one of the important components of inland water resources, the numerous lakes in China, as well as their responses to climate changes including lake area and lake level changes, have attracted widespread attention in recent years^12^. However, few studies have evaluated the long-term changes of lake water temperature under the scenario of rapid climate warming over China.

Since direct measurements of lake temperature are limited to only a few lakes, satellite-based thermal infrared (TIR) observations are often used for assessing LSWT variations at fine spatial and temporal scales. Various remote sensing platforms including Terra/Aqua-MODIS, Terra-ASTER, NOAA-AVHRR, ATSR and the Landsat series can provide worldwide observations of earth surface temperature^13,14^. To trace both the short-term and long-term variations of LSWT, moderate resolution imaging spectra-radiometer (MODIS) with daily collection of thermal images are used in this study. Several studies have investigated the variations of surface water temperature over individual large lakes, e.g., Siling Co^15^ and Lake Tai^16^ or lake zones, e.g., Tibetan Plateau^17,18^. However, such research efforts are relatively few in number and mainly limited to those alpine lakes with an average elevation of more than 4000 m above sea level. Given the increasing vulnerability of these lake resources to climatic and anthropogenic impacts, understanding the thermal behavior of lakes across China as well as the responses to climate warming is an issue of increasing concern.

In this study, we investigated the LSWT variations of large lakes in China by processing complete MODIS land surface temperature (LST) images over the country from 2001 to 2016. The temporal (diurnal, intra-annual and inter-annual) variations of LSWT as well as the spatial patterns across different lake zones were analyzed. We also evaluated the quantitative relationship of the lake temperature pattern with the geographical location, topography, and lake morphometric parameters. Correlation analyses between lake surface temperature and the surrounding air temperature were performed based on all the lakes in this study.

## Materials and methods

### Study area

**Figure 1:**
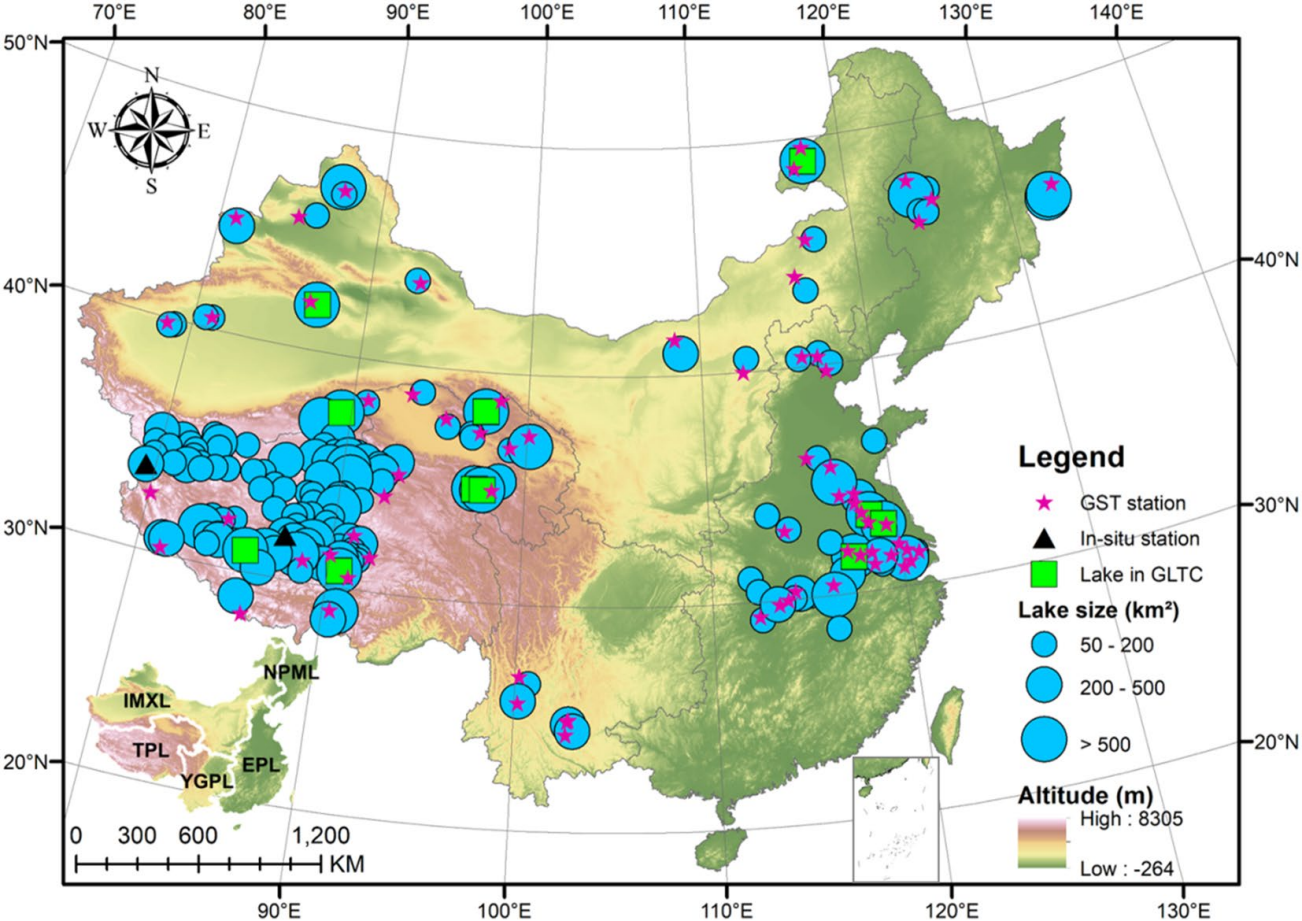
Distribution of the selected lakes in China, including the five lake-zones: Northeast Plain and Mountain Lake (NPML), Inner Mongolia-Xinjiang Lake (IMXL), Tibetan Plateau Lake (TPL), Eastern Plain Lake (EPL), and Yunnan-Guizhou Plateau Lake (YGPL). The five lake zones are highlighted at the bottom-left corner. Red stars are the location of ground surface temperature (GST) at 0 cm measured over 69 weather stations. Black triangles represent the location of field temperature measurement stations (Bangong Co and Dagze Co). The green rectangles indicate the 11 overlapped lakes in the Global Lake Temperature Collaboration (GLTC). Maps were generated in ArcMap software (Version 10.1; copyright and licensed by ESRI https://desktop.arcgis.com/).

China has a great spatial extent (75°–135° E, 18°–53° N) with a great wealth of lake resources over a large climatic range from the tropical to subarctic/alpine^9^. The numerous lakes are unevenly distributed across China, with a large cluster of alpine lakes on the Tibetan Plateau and abundant freshwater lakes across the Yangtze Basin. The distributions and abundance of these lakes are highly sensitive to climate variation and anthropogenic activities. The continuous climate warming over China in recent decades has had a significant impact on China’s water resources and agriculture^19^. Since the country’s highly complicated climate and topography, the lake systems have been strongly influenced by the diverse natural and human factors among different regions. For instance, drastic changes have occurred in the lakes across the Yangtze Basin as a result of excessive human activities, e.g., agricultural irrigation, water diversion projects, and land use changes^20,21^. In contrast, the Tibetan Plateau has witnessed rapid expansion of the alpine lakes over the past few decades due to accelerated glacier/snow melting and permafrost degradation^22^. According to the regional climates, geographical conditions, and socioeconomic factors, China’s lakes can be subdivided into five zones (Fig. 1): Northeast Plain and Mountain Lake (NPML), Inner Mongolia-Xin Jiang Lake (IMXL), Tibetan Plateau Lake (TPL), Eastern Plain Lake (EPL), and Yunnan-Guizhou Plateau Lake (YGPL)^9^.

### Data sets

#### MODIS LST data and lake boundary data

MODIS/Terra 8-day composite products (version 6) with a nominal pixel spatial resolution of 1 km (actual 0.927 km) at nadir (MOD11A2) from 2001 to 2016 were obtained from NASA’s Earth Observing System Data and Information System (EOSDIS, https://earthdata.nasa.gov). The MODIS/Terra LST data recorded at approximately 10:30 A.M. (10:30 P.M.) local time were obtained from brightness temperature utilizing a generalized split-window algorithm^23^. Brightness temperature or radiance temperature is the temperature of a blackbody that would emit the same amount of radiation as the targeted body in a specified spectral band^24^. The MODIS 8-day composite data have 46 8-day samples per year, which are the averaged LSTs of the MODIS daily products (MOD11A1) over the period of 8 days.

The surface water extent of lakes (area > 50 km^2^) across China was delineated from the global surface water dataset, which maps the extent and changes of surface water on a monthly time scale between 1984 and 2015 based on Google Earth Engine (https://earthengine.google.com/)^25^. This dataset has a satisfactory accuracy of water extraction, with the omission errors less than 5% and commission errors less than 1%. The lake boundary dataset for 2000, 2005, 2010, and 2015 were used to extract LSWT from the MODIS LST data between 2001 and 2016. To reduce the effect of water fluctuation along the shoreline, we excluded LST pixels within a 2-km buffer zone of the shoreline for each lake^15^. The lakes that fluctuate dramatically between the wet and dry seasons (e.g., Poyang Lake and Dongting Lake) were also excluded to reduce potential errors due to the intra-annual fluctuation of land–water interfaces. Following the above steps, the boundary polygons for a total of 169 lakes were finally obtained in this study.

#### Model validation datasets

At present, there are only a few sporadic field measurements of surface water temperature for lakes in China. Limited surface temperature data are available for two lakes in the Tibetan Plateau (Fig. 1), i.e., Bangong Co and Dagze Co (https://www.tpedatabase.cn/portal/MetaDataInfo.jsp?MetaDataId=249431). Bangong Co (78°25′–79°56′ E, 33°26′–33°58′ N) is the largest freshwater lake (salinity of 0.47 g/L) in the western Tibetan Plateau. The lake surface area is 627 km^2^, with the maximum depth of 41.7 m. Dagze Co (87°25′–87°39′ E, 31°49′–31°59′ N) is a salt-water lake (salinity of 14.69 g/L) in the central Tibetan Plateau. The surface area of Dagze Co is 245 km^2^, with the maximum depth of 38 m. Hourly measurements of water temperature of the two lakes were obtained using water temperature loggers^26^. The field measurement data of surface water temperature recorded at 10:30 P.M. (local time) between August and November 2012 by the uppermost loggers at the two lakes were used to be compared with the corresponding MODIS LSWT data (also taken at 10:30 P.M.).

In addition, a publicly available lake temperature data product, i.e., Global Lake Temperature Collaboration (GLTC, https://www.laketemperature.org), was also used to validate the accuracy of the LSWT data in this study. The GLTC data assembled a global database of summer lake temperatures for 291 lakes collected in situ and/or by satellites for the period 1985–2009^27^. The validation of the GLTC data was conducted by comparing surface temperature values for the 11 lakes that had both satellite and in situ data, which showed that the root mean square error (RMSE) value was 1.15 °C. There are eleven lakes in both the GLTC data set and the lake temperature data presented in this study during the overlap period of 2001–2009 (Fig. 1).

To further validate the reliability of MODIS LST data, we compared the MODIS LST time series values used in this study with ground surface temperature (GST) at 0 cm measured over 69 weather stations near to the mapped lakes distributed across China (Fig. 1). As reported in the previous studies^28^, the quality of MODIS LST product over lakes is expected to be better than that over the surrounding land surfaces, because lake surface water is relatively homogenous and the temporal variations of LSWT are more stable than that of land due to the larger heat capacity and less varied emissivity. Therefore, we collected the GST measurements over 69 weather stations from the China Meteorological Data Sharing System (CMDSS). The MODIS LST values over each station were estimated by averaging the pixel values within a 3 × 3 window centering the station. Daily average GST measurements were aggregated to 8-day average values according to the 8-day composite period of MODIS LST data.

#### Climatic data and lake morphometric parameters

Air temperature over each lake was obtained from a high resolution annual gridded temperature dataset (1 km resolution), which was provided by Data Center for Resources and Environmental Sciences, Chinese Academy of Sciences (RESDC) (http://www.resdc.cn). The lake surface elevations used in this study were derived from the Shuttle Radar Topography Mission (SRTM) digital elevation model (DEM) data at 90 m spatial resolution. Lake morphometric variables (e.g., surface area, mean depth, and volume) have an important influence on the surface water temperature of lakes^29^. The surface area of each lake is calculated using the lake boundary dataset developed in this study based on ArcGIS software. The mean depth and volume were derived from the HydroLAKES database which was developed by the Global HydroLAB (http://wp.geog.mcgill.ca/hydrolab/)^30^.

### Methods

The MODIS LST images were resampled to a 1 km pixel size and reprojected to an Albers Equal-Area Conic projection with the nearest neighbor interpolation method using MODIS Reprojection Tool (MRT, Land Processes DAAC, 2008). The LST images were then mosaicked and converted to a GEOTiff format. Four image layers were extracted from the MODIS LST product, including daytime LST, nighttime LST, and corresponding quality control (QC) images. The MODIS QC images contain the data quality flags which describe the quality of the retrieved LST pixels^31^.

To exclude problematic pixels in the LST images due to cloud contamination, the QC images and median filtering method were used in this study^32^. According to the data quality flag recorded in the QC images, all pixels with average LST errors less than 1 K (i.e., QC = 0, 1, 5, 17, 21) and pixels marked as good data quality (i.e., QC = 64 and 65) were retained, and pixels with other QC values were removed. A simple linear interpolation method was then used to fill the gaps of the MODIS 8-day composite data. To further reduce abnormal LST pixels, a median filtering method was used in the LST image sequence^18^. Overall, the MODIS 8-day composite data contains more than 70% valid pixels on average, and only 37 out of 1472 images (~ 2.5%) in the past 16 years contain less than 70% valid pixels.

In this study, the LSWT indicates the surface temperature for both the water and ice cover, and the ice surface temperatures derived from MODIS were used as the lake surface temperature in frozen periods^33^. The presence of ice cover may influence the temperature and circulation patterns of both the atmosphere and the lakes. Although the physical properties of the lake surface (surface temperature, albedo and roughness) are different with the presence of ice cover^34^, the MODIS provides an excellent measurement of the actual temperature of the surface of the ice cover^35^. For example, the MODIS ice surface temperature (IST) products are useful for determination of sea ice extent and ice surface temperature. Validations over different regions show a high accuracy for the MODIS-derived IST measurement with RMSE of 1.2–1.3 °C^35^.

The mean lake temperature during each 8-day composite period is the average value of the corresponding daytime temperature and nighttime temperature of that period. The mean temperature of each lake was calculated as the average value of all the pixels of daytime or nighttime LST images in the buffering zone of a given lake. The buffering zone of a given lake used in this study indicates the mask of the lake surface area after excluding LST pixels along the shoreline zones. The annual, seasonal, and monthly mean lake temperature in the daytime or nighttime were then calculated over the period 2001–2016 by aggregating the MODIS 8-day composite data for each lake. To estimate the diurnal temperature amplitudes for each lake, the DTDs of lakes were obtained by subtracting nighttime temperature from daytime temperature. In this study, the diurnal temperature range refers in particular to the temperature range between ~ 10:30 a.m. and ~ 10:30 p.m. local time. The full diurnal temperature range could be obtained by collecting both Aqua and Terra observations^36,37^. To investigate the temporal trend of surface temperature of each lake in both daytime and nighttime, the annual rates of temperature changes were estimated using a linear regression method:

$$ y = a + bx + e $$

where *y* denotes the annual mean temperature, *x* is the time series of years, *a* is the intercept, *b* is the rate of temperature change, *e* denotes the residuals; *a* and *b* are calculated using a least squares algorithm. The residual value was calculated as the difference between the observed temperature and the estimated temperature. The annual mean temperatures for different years are independent and not auto-correlated, and can be used to estimate the annual change rate of LSWT^17^. The linear regression method is commonly used to estimate the long-term trends of LSWT variations^17^.

To investigate the relative contributions of geographical location (latitude and longitude), topography (altitude), and lake morphometric factors (surface area, mean depth, and volume) on influencing the surface water temperature of lakes across China, stepwise multiple linear regressions were developed between lake surface temperature and the potential driving variables using SPSS Statistics software. Furthermore, the Pearson's correlation coefficients between the annual mean lake temperature and annual mean air temperature over each lake during the period 2001–2016 were also calculated in this study.

## Results and discussion

### Validation of MODIS LSWT data

**Figure 2:**
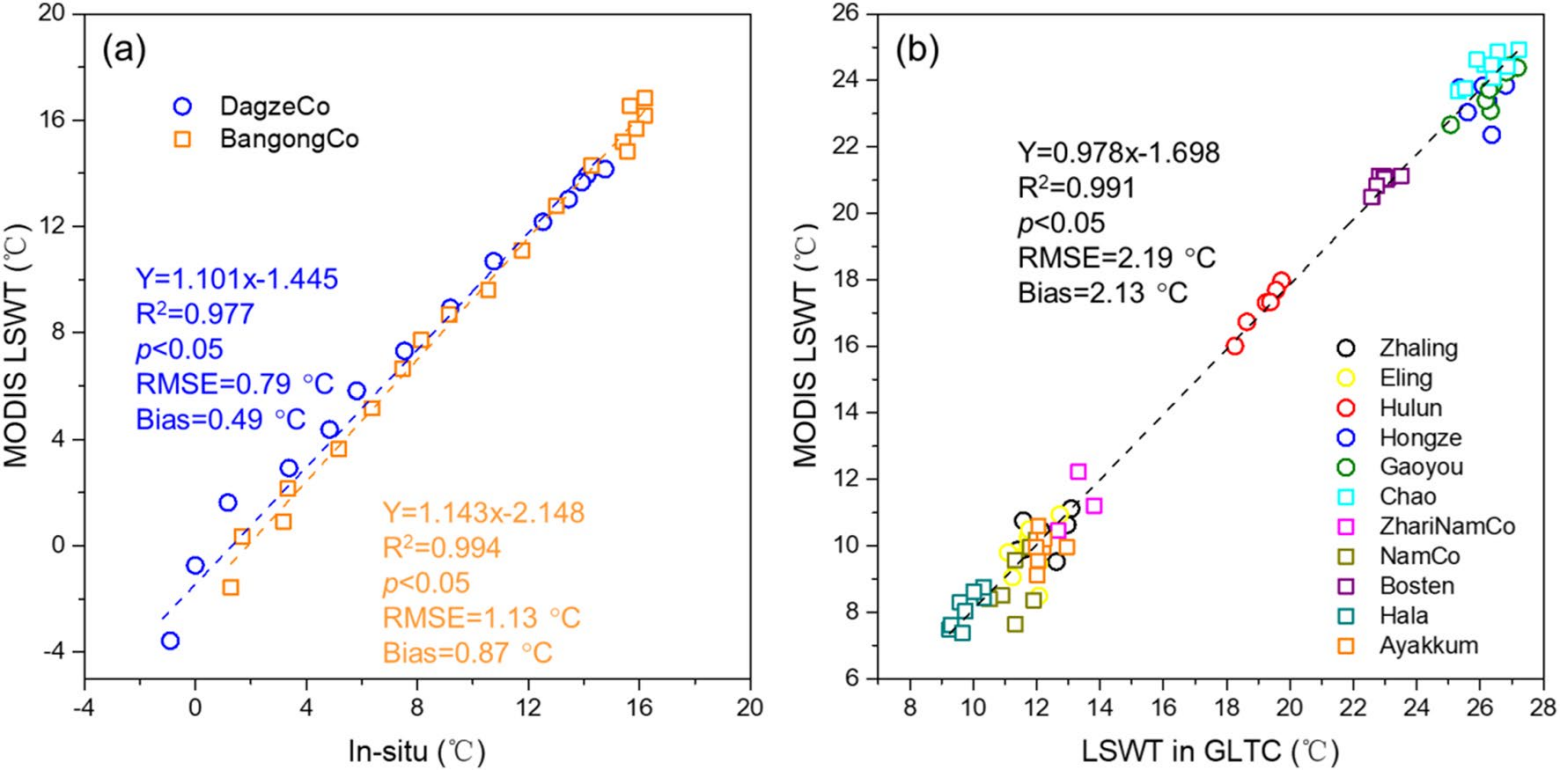
Comparisons of MODIS land surface water temperature (LSWT) product with (**a**) in situ measured data of Dagze Co and Bangong Co; and (**b**) GLTC data set for eleven lakes.

The in situ measured temperatures of Dagze Co and Bangong Co were collected to validate the MODIS LST product. The comparisons of MODIS LSWT with in situ measured lake temperature (Fig. 2) show a relatively high overall coefficient of determination (0.977 and 0.994) and low RMSE (0.79 and 1.13 °C), with a bias of 0.49 and 0.87 °C for Dagze Co and Bangong Co, respectively. The difference between MODIS LST products and in situ measurements could be attributed to many potential error sources, e.g., instrumental noise, cloud contamination, and land–water heat exchange effects^38^. Furthermore, the surface temperatures of the eleven lakes from the GLTC data set were used to compare with the MODIS lake temperature data generated in this study. The MODIS LSWT data agree well with the GLTC data (R^2^ = 0.991) with an average bias of 2.13 °C, with GLTC values higher than MODIS values. The deviation of the two data sets could be mainly caused by two factors, including the difference between the surface temperature (MODIS) and bulk temperature (GLTC), and the discrepancy of the satellite sensors^39^.

**Figure 3:**
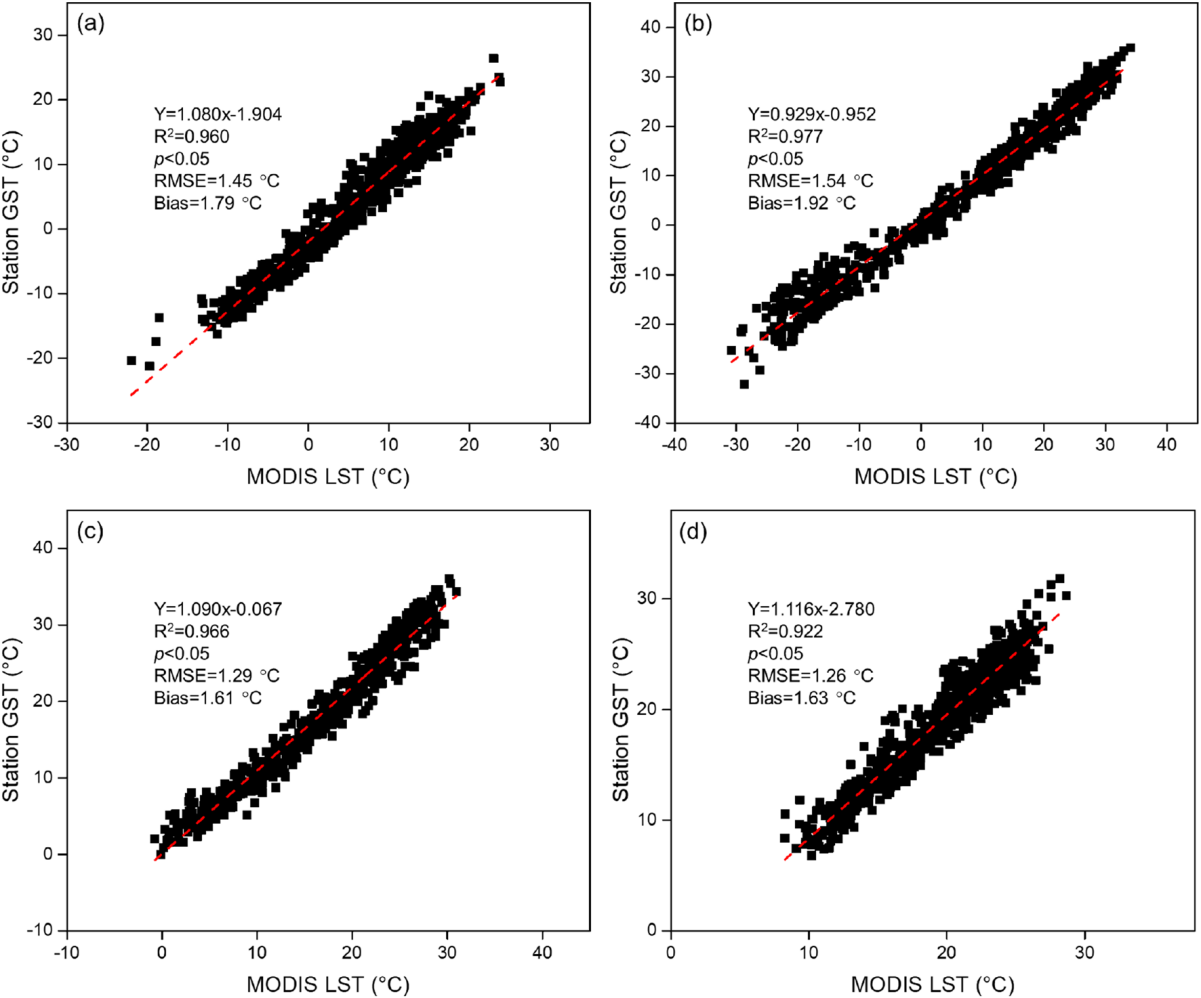
Comparison of 8-day mean MODIS LST with GST at the four weather stations near to (**a**) Lake Qinghai, (**b**) Lake Hulun, (**c**) Lake Taihu, and (**d**) Lake Dianchi, respectively.

The reliability of MODIS LST product was also validated by comparing MODIS LST time series values with the GST measurements of the whole day over the 69 weather stations near to the mapped lakes across China. Daily average GST measurements were aggregated to 8-day average values according to the 8-day composite period of MODIS LST data. The regressions between MODIS LST data and GST measurements were then conducted for each weather station. Figure 3 shows the scatterplots of all available 8-day mean MODIS LST during 2001–2016 against the corresponding GST measurements at the four weather stations near to Lake Qinghai, Lake Hulun, Lake Taihu, and Lake Dianchi, respectively. The four stations all show good consistency between MODIS derived LST data and GST measurements (R^2^ = 0.922–0.977, RMSE = 1.26–1.54 °C, and bias = 1.61–1.92 °C). Overall, relatively high coefficient of determination (average R^2^ = 0.963) and low average RMSE (1.79 °C) were achieved for all the weather stations, with average bias of 1.45 °C.

### Spatial pattern of LSWT

**Figure 4:**
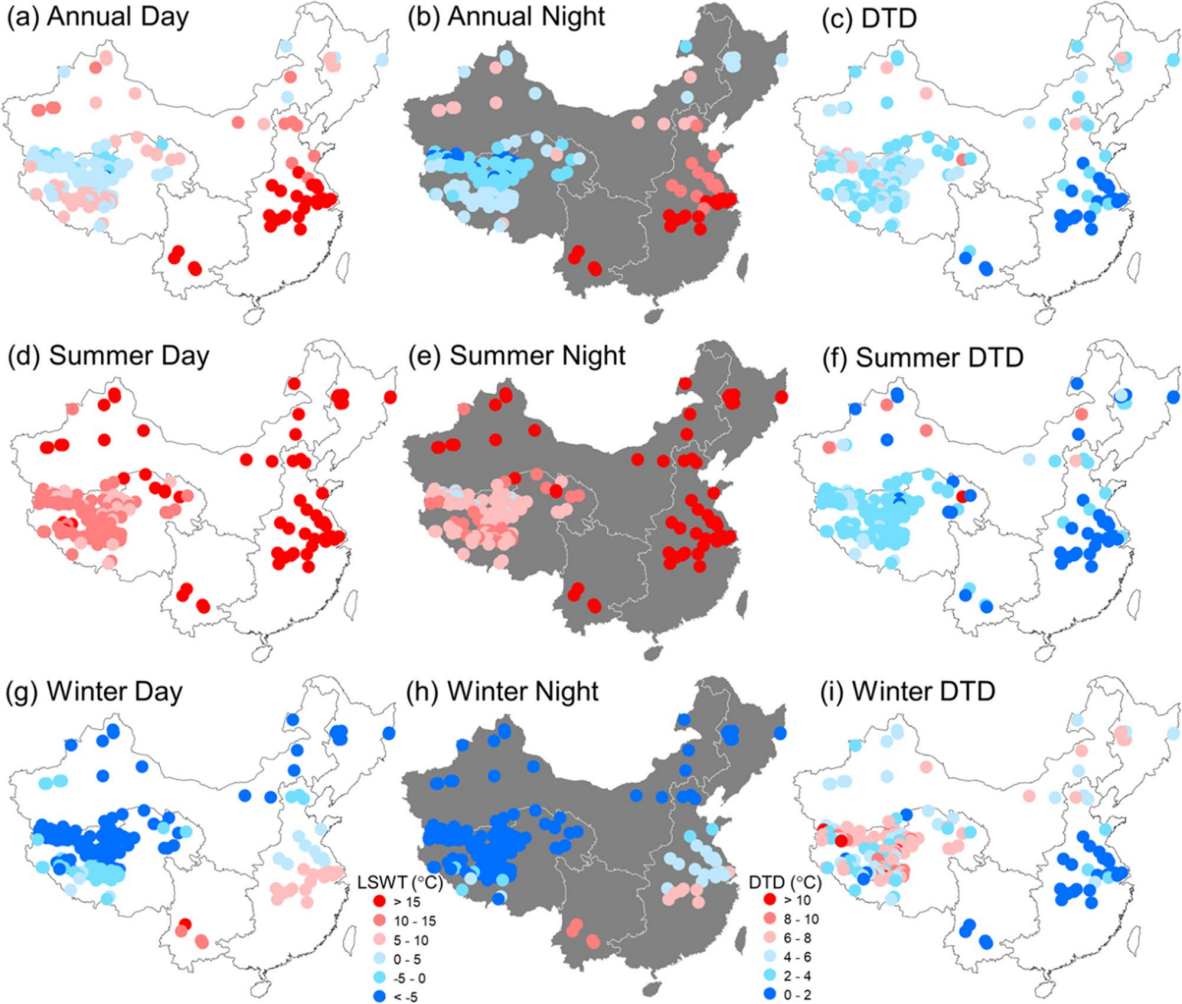
Spatial distributions of average surface water temperature of China's lakes over the period 2001–2016, including (**a**) daytime LSWT, (**b**) nighttime LSWT, (**c**) diurnal temperature difference (DTD), and their seasonal counterparts during summer (**d**–**f**) and winter (**g**–**i**), respectively. Maps were generated in ArcMap software (Version 10.1; copyright and licensed by ESRI https://desktop.arcgis.com/).

The spatial distributions of average surface water temperature of China's lakes over the period 2001–2016 were investigated (Fig. 4). The annual LSWT averaged from 2001 to 2016 demonstrated a large spatial variation in the lakes across China varying from − 2.26 to 19.56 °C for daytime and − 9.01 to 17.16 °C for nighttime, respectively. Overall, warmer lakes were located in the EPL and YGPL zones, while relatively low-temperature lakes were mostly situated in the TPL zone (Fig. 4a,b). Moreover, the annual mean DTDs also exhibited a distinct spatial pattern (Fig. 4c). Small DTDs were observed for the lakes in the EPL zone, and large DTDs were generally found for the lakes located in the TPL zone. Combination of both daytime and nighttime LSWT patterns indicates that large and deep lakes with huge water storage showed less variability for DTDs, i.e., Lake Tai, Lake Chao, and Lake Hong in the EPL zone, and Lake Qinghai, Nam Co, and Siling Co in the TPL zone. The spatial patterns of LSWT differed greatly by season. In summer, the mean LSWT values were over 15 °C in the lakes of all regions except the TPL (Fig. 4d,e). In contrast, the mean LSWT values were negative in the lakes of all regions except EPL and YGPL during the winter (Fig. 4g,h). The seasonal DTDs also showed significant difference between summer and winter, especially in the TPL zone. In winter, the large DTDs were found in the lakes of the TPL, which may be a result of the fact that LSWT drops rapidly from late autumn to winter along with the occurrence of ice cover, and the lake surface has a large variation of temperature between daytime and nighttime due to the low specific heat capacity of ice^15,18^.

### Temporal variations of LSWT

#### Intra-annual variations of LSWT

**Figure 5:**
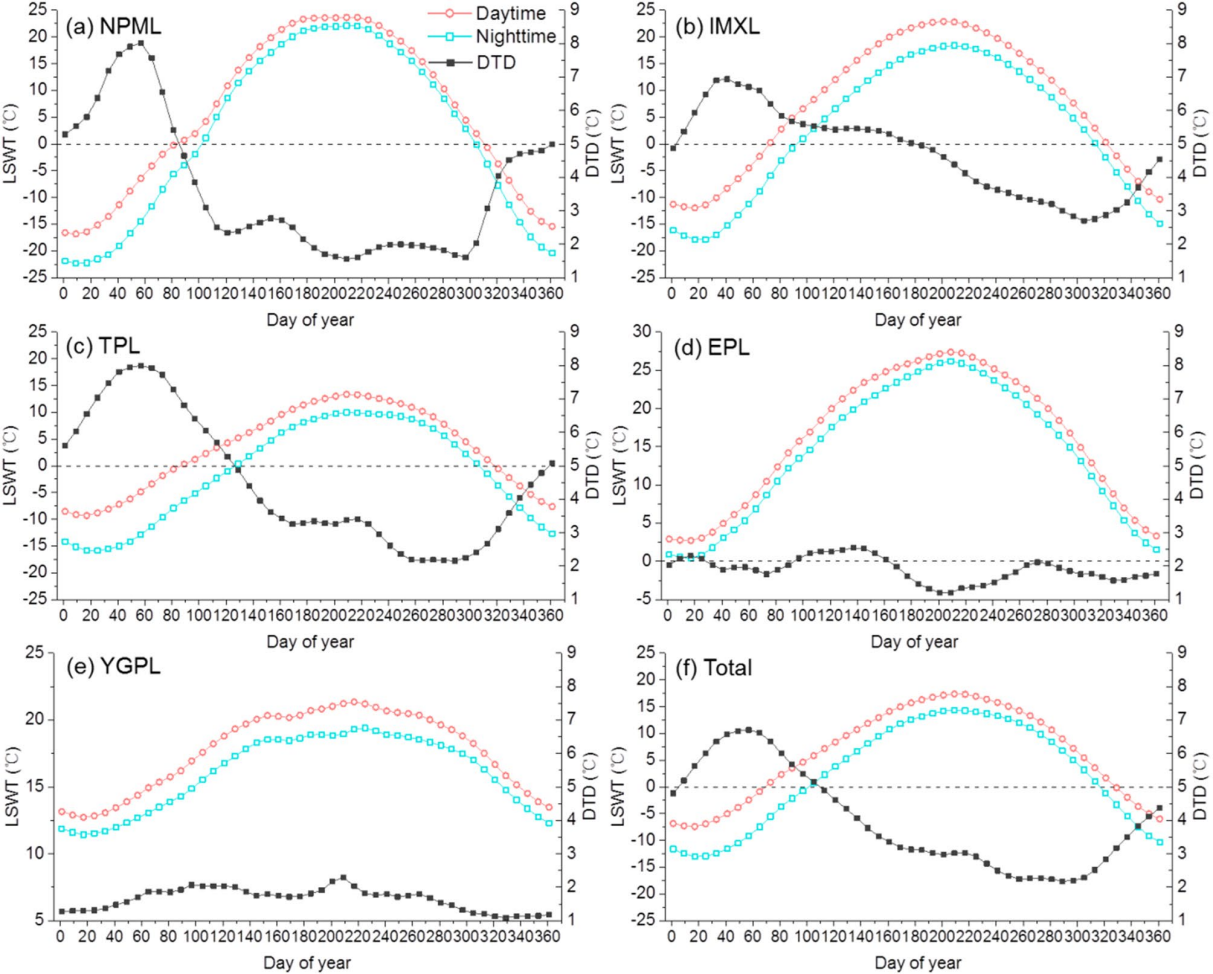
16-year average water surface temperature for daytime, nighttime, and the diurnal temperature differences (DTDs) through the year for the lakes in (**a**) NPML, (**b**) IMXL, (**c**) TPL, (**d**) EPL, (**e**) YGPL, and (**f**) the whole country.

We investigated the variations of average LSWT over the period 2001–2016 for daytime, nighttime, and DTDs throughout the year (Fig. 5). The overall temperature of both daytime and nighttime (Fig. 5f) gradually increases from the minimum in late January (Julian day 17–25) to the maximum in late July (Julian day 201–217). The daytime temperature was about 0 °C in late March (Julian day 81), while the nighttime temperature reached 0 °C in late April (Julian day 113). The average overall lake temperature drops to 0 °C in late November (Julian day 329) for the daytime and in the middle of November (Julian day 321) for the nighttime, respectively. Along with the expansion of ice cover on the lake, LSWT is continuously declining until the whole lake freezes up. For the temperature cycle of lakes, the beginning of ice cover break-up and the time of minimum and maximum water surface temperature are of paramount importance. The timing of lake ice cover freeze-up or break-up is an important parameter for biological and chemical process inside lakes^40^. The maximum DTD (6.7 °C) was recorded during the ice break-up period (Julian day 57, late February), gradually decreased until ice freeze-up occurs (2.2 °C), and then an increasing trend followed. The intra-annual variations of average LSWT and DTDs for the NPML, IMXL, and TPL regions followed a similar pattern, while the maximum value and the specific dates reaching the maximum DTD varied across different regions. The intra-annual variations of averaged LSWT and DTDs showed distinct patterns across different regions. Both daytime and nighttime LSWT in the EPL and YGPL zones were positive throughout the year, and the largest DTD was found in the middle of May (2.5 °C) and in late July (2.3 °C), respectively.

#### Inter-annual variations of LSWT

**Figure 6:**
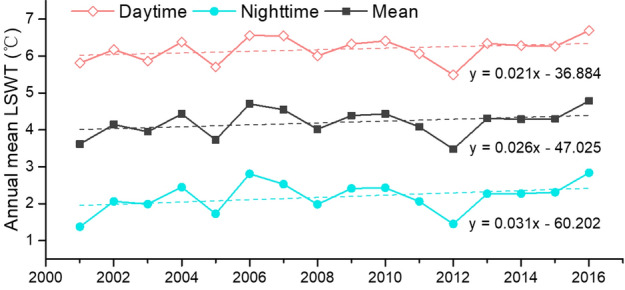
Inter-annual changes of annual mean daytime, nighttime and mean lake surface water temperature (LSWT) from 2001 to 2016.

The inter-annual variations of LSWT from 2001 to 2016 for both daytime and nighttime are investigated (Fig. 6). During the past 16 years, the annual mean daytime and nighttime temperature fluctuated coincidently, reaching the peak value in 2006 and declining to the valley in 2012. Overall, the annual average LSWT increased at a rate of 0.26 °C/decade. The increase rate of annual mean nighttime temperature is about 0.31 °C/decade, faster than that of daytime temperature (0.21 °C/decade). The results confirm previous research which has also shown that the processes responsible for the long-term variations in the temperature structure of the lake appeared mainly during the nighttime^41^. Existing studies with regard to climate change over the TPL zone reported that the rapid increase in lake temperature of the nighttime might be largely due to the increasing low-level cloud cover at nighttime^42^.

**Figure 7:**
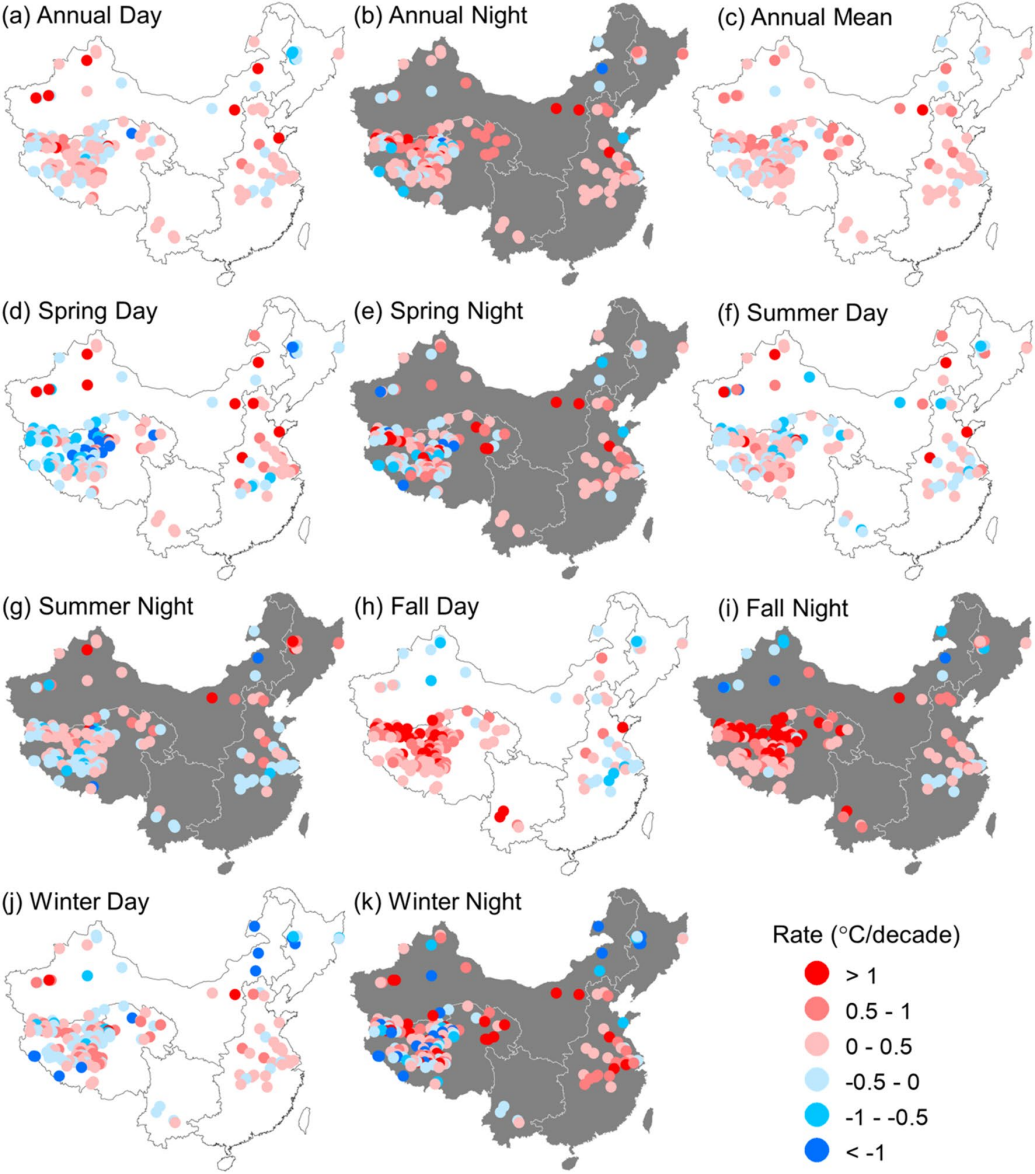
Spatial patterns of lake surface water temperature (LSWT) change rates including annual mean daytime LSWT (**a**), nighttime LSWT (**b**), mean LSWT (**c**), and the seasonal variations during spring (**d** and **e**), summer (**f** and **g**), fall (**h** and **i**), and winter (**j** and **k**), respectively. Maps were generated in ArcMap software (Version 10.1; copyright and licensed by ESRI https://desktop.arcgis.com/).

The spatial distributions of LSWT change rate (°C/decade) from 2001 to 2016 are demonstrated in this study (Fig. 7). The daytime and nighttime trends for each lake exhibited various patterns, and only 23 lakes showed consistent temperature trends in both daytime and nighttime during the past 16 years. According to the daytime LSWT measurements, 121 (71.6%) lakes showed a gradual increase in temperature with a mean rate of 0.38 °C/decade, while the rest 48 (28.4%) lakes decreased in temperature with a mean rate of − 0.21 °C/decade. Regression analysis indicated that the daytime LSWT trend of 30 lakes is significant (*P* < 0.1), including 28 warming and 2 cooling lakes. With respect to nighttime MODIS LST measurements, 134 (79.3%) lakes increased in temperature with a mean rate of 0.48 °C/decade, and 35 (20.7%) lakes decreased in temperature with a mean rate of − 0.35 °C/decade. The change trend in surface temperature of 40 lakes was statistically significant (*P* < 0.1), including 37 warming and 3 cooling lakes for nighttime. The inter-annual variations of LSWT showed significant difference among different seasons (Fig. 7d–k). The increasing trends of LSWT for both daytime and nighttime were more intense in fall than in other seasons. Statistics on change rates of seasonal average temperature showed that a remarkable increase of average LSWT occurred in fall, with a rising rate of 0.43 °C/decade at daytime and 0.55 °C/decade at nighttime. Spatially, notable increasing trends of LSWT were observed in the TPL region in fall day and fall night. The change rates of LSWT in spring night and winter night were relatively higher in the EPL region than those in other regions.

### Relationships between LSWT and potential drivers

To explore the relative importance of geographical location, topography, and lake morphometric factors in influencing the LSWT across China, multivariate regression analysis was conducted based on SPSS Statistics software. Six factors including latitude, longitude, altitude, lake surface area, depth, and volume were selected as possible explanatory variables in this study. The geographical location (represented by latitude and longitude) was used to evaluate the effects of the solar radiation and air temperature, and the topography (indicated by altitude) was referred to as the topographic impacts on LSWT. The influence of morphological factors (including lake surface area, depth, and volume) on LSWT was also estimated. Multivariate regression model was constructed by regressing the mean surface water temperature on the six explanatory variables for all the lakes. The stepwise forward selection method was utilized to develop the linear regression model, and the predictor or input variable with statistical significance (α < 0.05) was reserved in the model. The multivariate regression analysis indicated that the four variables (altitude, latitude, longitude, and volume) could explain a large proportion of the variance in the LSWT (Table 1), which is statistically significant as indicated by R^2^ = 0.972 and *P* < 0.001. The significant negative relationship between LSWT with the altitude showed the topographical effect on LSWT, and the alpine lakes at high altitude have relatively low LSWT in both daytime and nighttime. The regression model indicated that LSWT might decrease by 0.557 °C in daytime or 0.592 °C in nighttime when the latitude increased by 1°. There is also a negative relationship between LSWT and longitude, and LSWT may decrease by 0.283 °C in daytime or 0.242 °C in nighttime when the longitude increases by 1°. Moreover, the lake volume has a positive effect on the lake temperature in nighttime. Overall, geographical location and topography are more significant factors in explaining the national lake water temperature variation than the morphological factors. Previous studies have also suggested that the variations of lake thermal pattern were closely related to the climatic factors including absorbed solar irradiance and heat exchange with the atmosphere^43^, but were also mediated by lake morphology such as surface area, depth, and volume^5^.

**Table 1:** Stepwise multivariate linear regression models for predicting lake surface water temperature (LSWT) based on all lakes.

Dependent variable	Independent variables	Coefficient	Standard error	Partial *p*-value	Model *p*-value	VIF	R^2^
LSWT (day)	Intercept	53.110	1.843	0.000	0.000		0.948
	Altitude	− 1.258	0.000	0.000		3.613	
	Latitude	− 0.557	0.023	0.000		1.162	
	Longitude	− 0.283	0.013	0.000		3.357	
LSWT (night)	Intercept	57.355	1.767	0.000	0.000		0.966
	Altitude	− 1.230	0.000	0.000		3.620	
	Latitude	− 0.592	0.023	0.000		1.167	
	Longitude	− 0.242	0.013	0.000		3.357	
	Volume	0.071	0.000	0.002		1.015	
LSWT (mean)	Intercept	55.276	1.488	0.000	0.000		0.972
	Altitude	− 1.252	0.000	0.000		3.620	
	Latitude	− 0.580	0.019	0.000		1.167	
	Longitude	− 0.261	0.011	0.000		3.357	
	Volume	0.045	0.000	0.000		1.015	

To further analyze the possible impacts of the six explanatory variables on diurnal lake temperature variation, the multivariate regression model was also constructed based on all the lakes. As illustrated in Table 2, the multivariate regression model is of statistically significance with a moderate coefficient of determination (R^2^ = 0.426, *P* < 0.001). The three variables (altitude, latitude, and lake surface area) play an important role in explaining the variance in the DTDs. The DTDs are positively correlated with the altitude and the diurnal amplitude in LSWT is expected to be greater with the increase in the elevation of lakes, which showed the topographical effect on the DTDs. There also exist positive relationships of DTDs with the latitude, and dramatic temperature differences between day and night are generally found for the lakes at high latitude. In addition, the DTDs are negatively correlated with the lake size, and the lakes with relatively small water area would have large variations in DTDs due to low heat storage capacity.

**Table 2:** Stepwise multivariate linear regression models for predicting diurnal temperature difference (DTD) based on all lakes.

Dependent variable	Independent variables	Coefficient	Standard error	Partial *p*-value	Model *p*-value	VIF	R^2^
DTD	Intercept	− 4.404	0.970	0.000	0.000		0.426
	Altitude	0.566	0.000	0.000		1.167	
	Latitude	0.495	0.025	0.000		1.162	
	Area	− 0.257	0.000	0.000		1.033	

The variations of the lake thermal characteristics integrate multiple factors related to regional climate through heat exchange with the surrounding atmosphere. The response of lake surface temperature to environmental changes is not only controlled by climatic factors including air temperature, solar radiation, humidity, ice cover, and wind^43^, but also the lake-related parameters, e.g., lake bathymetry, water clarity, and river inflows^44^. Some pioneering studies on the thermal behavior of lakes have reported that climatic factors (latitude, longitude, and altitude), lake morphometric parameters (surface area, depth, volume), and water quality factors could affect the lake temperature through their impacts on the lake-atmosphere heat interactions^45^. However, the knowledge about the quantitative impacts of these factors on the lake thermal variability across China is still limited. In this study, the results can provide a further understanding of the response of lake skin temperature to the various factors under the scenario of climate warming.

### Comparisons between inter-annual trends of LSWT and air temperature

**Figure 8:**
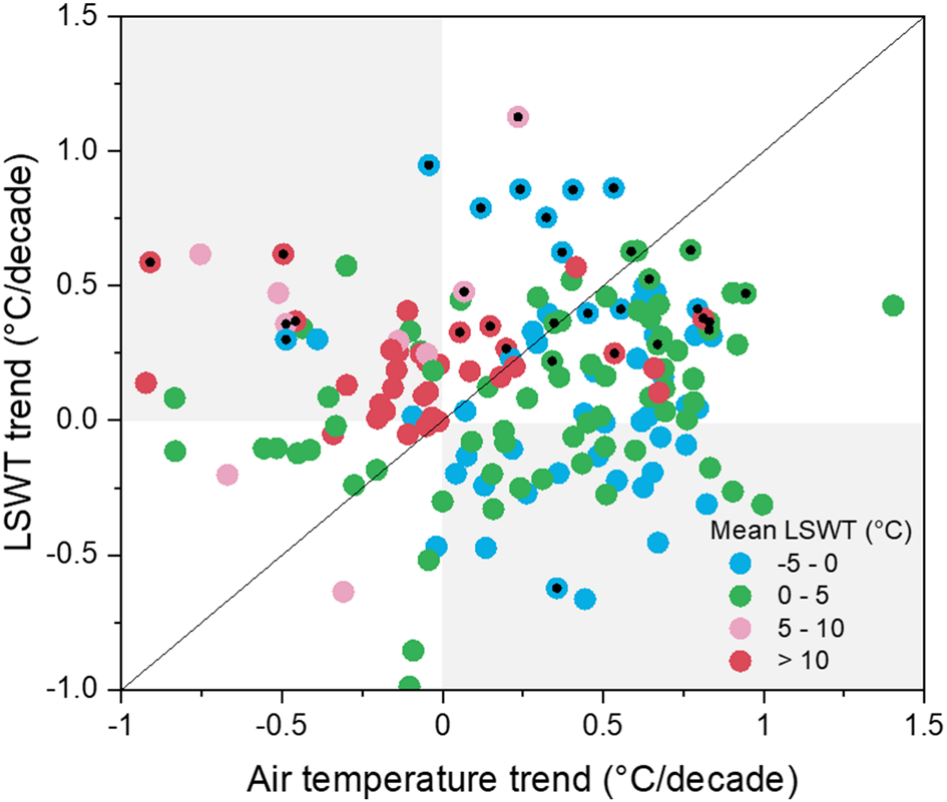
Comparison between LSWT trend and air temperature trend for all lakes during the period 2001–2016. Significant trend (*P* < 0.1) of the annual average LSWT in lakes are indicated by a black central dot within a data point.

**Figure 9:**
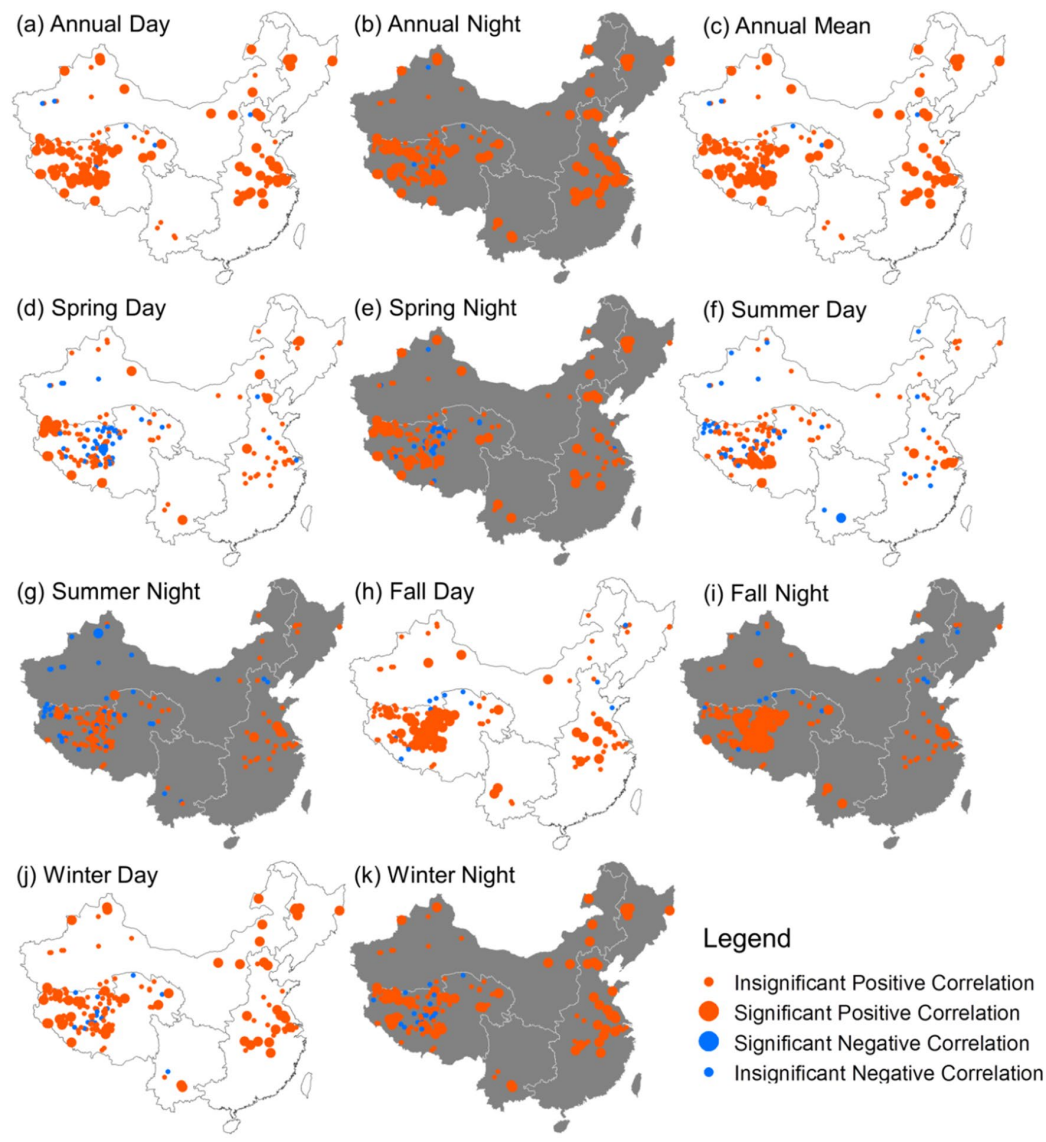
Pearson’s correlation analyses between LSWT and air temperature in each lake during 2001–2016. Maps were generated in ArcMap software (Version 10.1; copyright and licensed by ESRI https://desktop.arcgis.com/).

For each individual lake, we used a linear regression model to calculate trends in annual average LSWT and air temperature. Pearson's correlation analyses were conducted based on SPSS Statistics software to examine the relationships between LSWT trend and air temperature trend for each lake. The results showed that the lake surface temperature has been warming significantly, with a mean trend of 0.26 °C/decade, across all the lakes between 2001 and 2016 (Fig. 8). This warming rate is consistent with the rapid increase in annual average air temperatures (0.26 °C/decade) during the study period. The patterns of climate warming and lake temperature variations were highly variable, and the difference between the overall trend for air temperature and LSWT was not statistically significant across these lakes (Fig. 8). Pearson's correlation analyses between LSWT and air temperature during 2001–2016 for all the lakes were also investigated (Fig. 9). The LSWT was positively correlated with air temperature in both daytime and nighttime for most lakes (over 62.7% of lakes showed significant positive correlations). There are significant positive correlations between the LSWT and air temperature in most lakes in fall and winter for both daytime and nighttime. Previous studies have suggested that large inland water bodies worldwide showed similar warming trends with the independent air temperature observations^5,6,46^. This suggests that satellite-derived lake surface temperature could reflect the variations of regional air temperature and can thus serve as an excellent indicator of climate change^4,47^.

## Conclusion

In this study, the spatial and temporal variations of LSWT of China’s lakes were investigated by employing MODIS LST data from 2001 to 2016. The temporal (diurnal, intra-annual and inter-annual) variations of LSWT as well as the spatial patterns across different lake zones were analyzed. The daytime and nighttime trends of the lake skin temperature variations exhibited various patterns. Overall, 121 (71.6%) lakes showed an increase in daytime temperature with a mean rate of 0.38 °C/decade, while the rest 48 (28.4%) lakes decreased in temperature with a mean rate of − 0.21 °C/decade. We also evaluated the relationship of the lake thermal pattern with the geographical location, topography, and lake morphometric parameters. The geographical location and topography were found to be the primary driving factors in explaining the national lake water temperature variations, which are also mediated by morphometric factors such as lake surface area and volume. In addition, Pearson's correlation analyses were conducted to examine the relationships between LSWT trend and air temperature trend for each lake. There are significant positive correlations between the LSWT and air temperature in both daytime and nighttime for most lakes (over 62.7%). The comprehensive analysis of the national-scale LSWT variations provides a better understanding of the response of lake thermal patterns to various climatic and geomorphic factors in China and useful information for future water resource monitoring under global warming.

## Data Availability

The relevant data in this study are available from the authors.
